# Regulation of the effector function of CD8^+^ T cells by gut microbiota-derived metabolite butyrate

**DOI:** 10.1038/s41598-018-32860-x

**Published:** 2018-09-26

**Authors:** Maik Luu, Katharina Weigand, Fatana Wedi, Carina Breidenbend, Hanna Leister, Sabine Pautz, Till Adhikary, Alexander Visekruna

**Affiliations:** 1 Institute for Medical Microbiology and Hygiene, Philipps University of Marburg, Marburg, Germany; 2 Department of Biochemistry, University of Kassel, Kassel, Germany; 3 Institute of Molecular Biology and Tumor Research (IMT), Center for Tumor- and Immunobiology, Philipps- University Marburg, Marburg, Germany

## Abstract

The gut microbiota produces metabolites such as short-chain fatty acids (SCFAs) that regulate the energy homeostasis and impact on immune cell function of the host. Recently, innovative approaches based on the oral administration of SCFAs have been discussed for therapeutic modification of inflammatory immune responses in autoimmune diseases. So far, most studies have investigated the SCFA-mediated effects on CD4^+^ T cells and antigen presenting cells. Here we show that butyrate and, to a lesser degree, propionate directly modulate the gene expression of CD8^+^ cytotoxic T lymphocytes (CTLs) and Tc17 cells. Increased IFN-γ and granzyme B expression by CTLs as well as the molecular switch of Tc17 cells towards the CTL phenotype was mediated by butyrate independently of its interaction with specific SCFA-receptors GPR41 and GPR43. Our results indicate that butyrate strongly inhibited histone-deacetylases (HDACs) in CD8^+^ T cells thereby affecting the gene expression of effector molecules. Accordingly, the pan-HDAC inhibitors trichostatin A (TSA) and sodium valproate exerted similar influence on CD8^+^ T cells. Furthermore, higher acetate concentrations were also able to increase IFN-γ production in CD8^+^ T lymphocytes by modulating cellular metabolism and mTOR activity. These findings might have significant implications in adoptive immunotherapy of cancers and in anti-viral immunity.

## Introduction

The short-chain fatty acids (SCFAs) acetate, propionate and butyrate are synthesized in the intestinal lumen of caecum and large intestine by bacterial fermentation of non-digestible, complex carbohydrates such as dietary fiber^1^. SCFAs are capable of crossing the intestinal epithelium and of reaching the lamina propria, where they can directly shape mucosal immune responses. A high intake of dietary fiber or oral administration of SCFAs have been shown to mediate protective effects in experimental models of colitis, multiple sclerosis, type 1 diabetes, allergic airway inflammation and food allergy^2–6^. Acetate, which is the most abundant SCFA in the intestinal lumen, has been shown to be an important substrate for hepatic lipogenesis. Propionate can also be metabolized in the liver acting as substrate for the hepatic gluconeogenesis. Butyrate, which is mainly produced by strictly anaerobic spore-forming bacteria such as *Clostridium butyricum*, is used locally as energy source for colonocytes^7^. Novel reports have found that butyrate and other SCFAs are able to interact with their specific receptor GPR43, which is selectively expressed on colonic regulatory T cells (Tregs)^8^. Alternatively, SCFAs taken up by mucosal T cells act directly in the nucleus as histone deacetylase (HDAC) inhibitors, thereby promoting differentiation of peripheral Tregs. This effect of butyrate on Tregs can be explained by increasing the acetylation of histones H3 and H4 at the *Foxp3* gene locus^9,10^. Taken together, SCFAs that are absorbed first into colonocytes and then into mucosal immune cells profoundly impact on intestinal homeostasis by inducing generation of Tregs, by enhancing the gut barrier function and by influencing signaling pathways that govern dendritic cells (DCs) to a tolerogenic state^7^.

While the anti-inflammatory capacity of butyrate and other SCFAs has been extensively investigated, novel studies have revealed that CD4^+^ effector T cells might also be a cellular target for SCFAs^11–14^. Therefore, it will be particularly interesting to better understand the molecular mechanisms underlying cell- and tissue-specific responsive immune cell subsets in order to develop and provide a safe SCFA-based therapy for patients with autoimmune diseases. Due to their HDAC-inhibitory activity and strong interaction with cell surface receptors such as GPR41, GPR43 and GPR109A, SCFAs have a strong potential to regulate the function of immune cells in extra-intestinal organs as well (particularly if administered intravenously or intraperitoneally). So far it has clearly been demonstrated that SCFAs are able to modulate the phenotype and function of numerous immunologically relevant cells such as colonic epithelial cells, macrophages, neutrophils and DCs^15–18^.

The unanswered question is if microbial metabolites are capable of regulating the gene expression and function of CD8^+^ T lymphocytes. Our current findings suggests a strong effect of butyrate on two CD8^+^ T cell subsets, cytotoxic T lymphocytes (CTLs) and Tc17 cells. Several lines of evidence point to epigenetic regulatory mechanisms causing effects of butyrate on CD8^+^ T cell function. Thus, our study supports the concept that SCFAs not only optimize the function of Tregs and conventional CD4^+^ T cells, but also modulate the expression of effector molecules in CD8^+^ T lymphocytes in a context-specific manner.

## Results

### Butyrate promotes the increased expression of IFN-γ and granzyme B in CTLs and Tc17 cells

**Figure 1:**
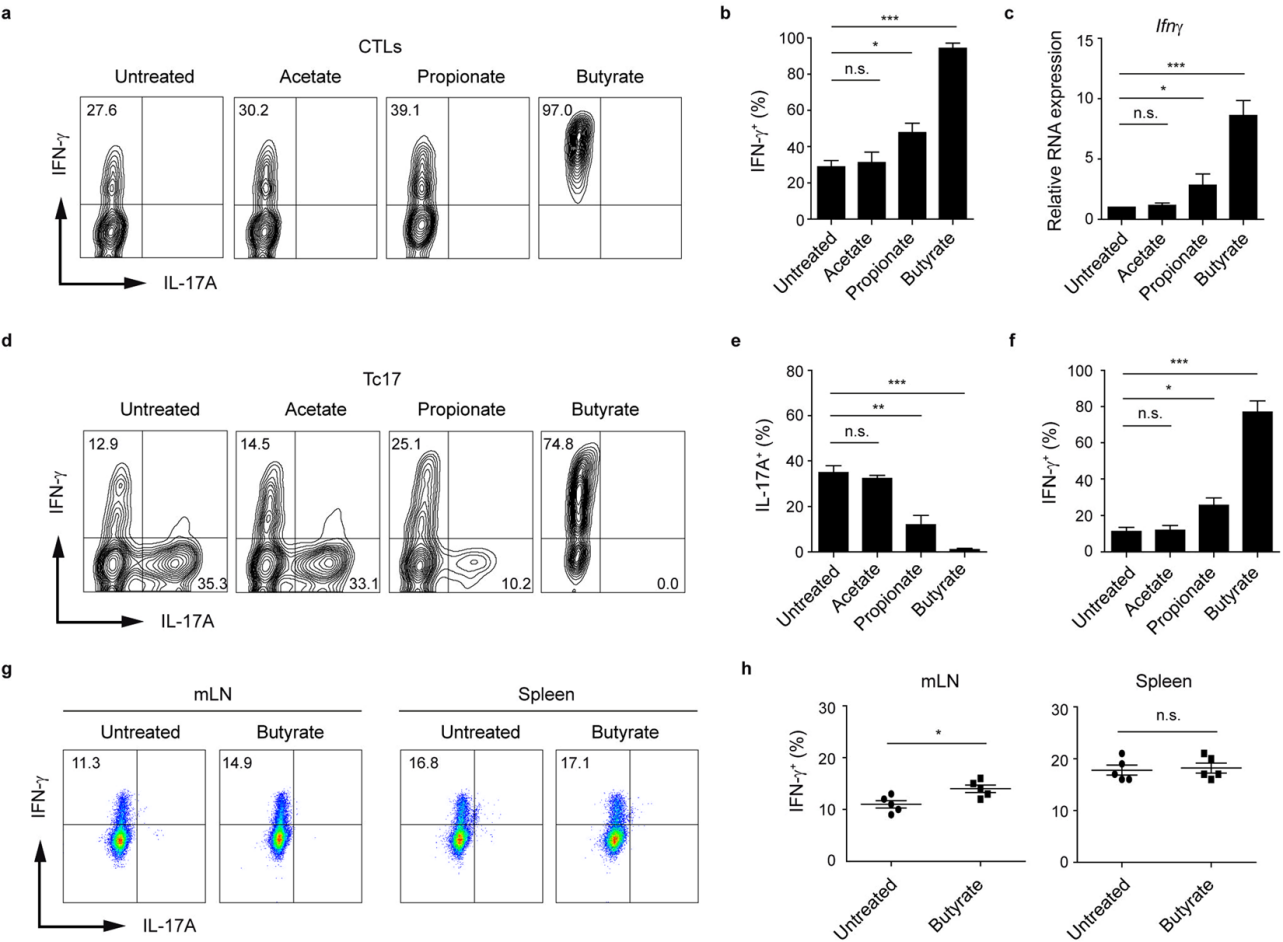
Treatment of CD8^+^ T cells with butyrate results in preferential increase of IFN-γ production. (**a**,**b**) Frequency of IFN-γ^+^ cells cultured under sub-optimal CTL-inducing conditions and treated with SCFAs (1 mM). (**c**) Relative mRNA expression of *Ifn*γ in CTLs treated with 1 mM of various SCFAs. (**d**–**f**) Frequencies of IFN-γ^+^ and IL-17A^+^ Tc17 lymphocytes upon treatment with SCFAs (1 mM). (**g**,**h**) Frequency of IFN-γ^+^ CTLs in mLNs and spleen four weeks after oral treatment of WT mice with 150 mM sodium butyrate. Two experiments with five mice per group were performed. (**b**,**c**,**e**,**f**) Data are pooled from three independent experiments. Results are expressed as mean ± SEM. n. s. = not significant, ^*^p < 0.05, ^**^p < 0.01, ^***^p < 0.001.

To investigate if SCFAs are able to influence the phenotype of CD8^+^ T cells, we treated CTLs and Tc17 cells with acetate, propionate and butyrate for three days and measured the expression of IL-17A and IFN-γ in both CD8^+^ T cell subsets by flow cytometry. As compared to acetate-treated or untreated T cells, the frequency of IFN-γ^+^ cells increased significantly following butyrate treatment of both, CTLs and Tc17 cells (Fig. 1a–f). Moreover, the reduction of IL-17A was detected in Tc17 cells treated with butyrate but not with acetate. Propionate treatment also led to increased percentages of IFN-γ^+^ cells, however this effect was less pronounced as compared to the treatment with butyrate. We next investigated whether *in vivo* treatment with butyrate could specifically alter IFN-γ production by CD8^+^ T cells. To test if IFN-γ production in CD8^+^ T cells might be upregulated by butyrate, WT mice were orally treated with this SCFA for three weeks (according to the published protocol^8^) and afterwards the frequency of IFN-γ-expressing CD8^+^ and CD4^+^ T cells in the spleen and mLNs was examined by FACS analysis. Intracellular staining revealed a slight, but reproducible increase in percentage of IFN-γ^+^ CD8^+^ T lymphocytes in mLNs but not in spleen (Fig. 1g,h). In contrast, CD4^+^ effector T cells derived from both organs were not able to increase their IFN-γ production after treatment with butyrate (Supplementary Fig. 1a,b). Negligible percentages of Th17 and Tc17 cells were measured in both tissues independently of butyrate treatment. Previously, it was demonstrated that early, autocrine IFN-γ signaling promotes the CTL cell differentiation and upregulates T-bet and granzyme B expression in CD8^+^ T cells^19^. It is tempting to speculate that butyrate could locally enhance already high production of IFN-γ in mLNs by supporting the autocrine IFN-γ signals.

**Figure 2:**
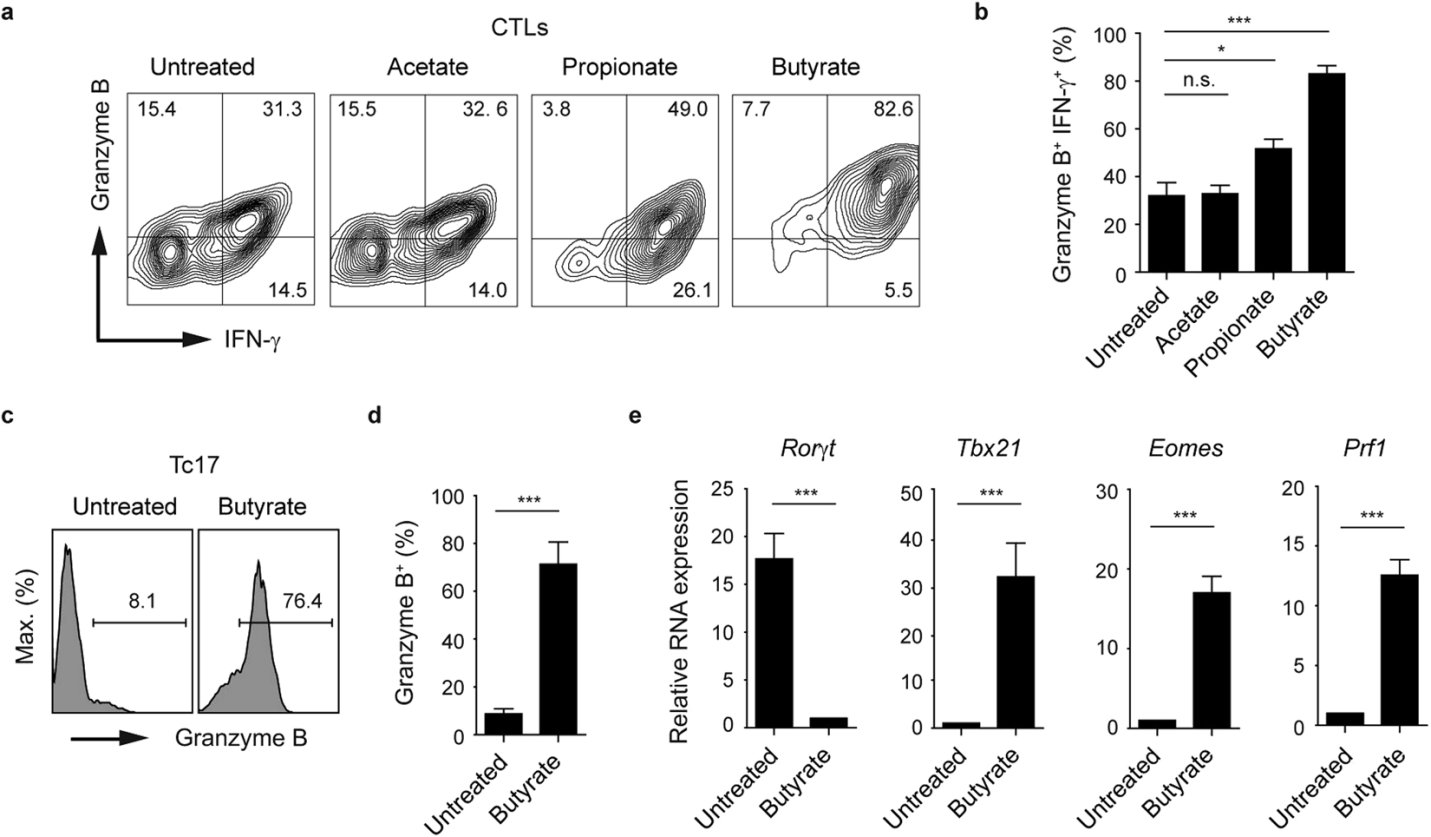
Effects of butyrate on the expression of CTL-related effector molecules. (**a**,**b**) Percentages of granzyme B^+^ IFN-γ^+^ CTLs in the presence of various SCFAs (1 mM). (**c**,**d**) Expression of granzyme B in Tc17 cells treated with 1 mM sodium butyrate for three days. (**e**) Quantitative RT-PCR analysis of *Ror*γ*t* and CTL-associated genes *Tbx21*, *Eomes* and *Prf1* in Tc17 cells treated with 1 mM sodium butyrate for two days. (**b**,**d**,**e**) Three independent experiments were performed. Results are shown as mean ± SEM. n. s. = not significant, ^*^p* < *0.05, ^***^p < 0.001.

To test whether CTLs are able to upregulate their granzyme B expression upon SCFA treatment, we treated CD8^+^ T lymphocytes with acetate, propionate and butyrate for three days, and measured the concurrent production of the CTL-associated effector molecules IFN-γ and granzyme B. While butyrate was capable of strongly upregulating the frequency of granzyme B^+^ IFN-γ^+^ CD8^+^ T cell population, no effect of acetate on CTLs was observed. Furthermore, a slight increase in percentages of double-positive population was detected after propionate treatment (Fig. 2a,b). To examine the stability of butyrate-mediated phenotype, we treated CTLs with butyrate for two days. Subsequently, butyrate was removed from cell culture supernatants and three days later the expression of IFN-γ was measured by flow cytometry. After 5 days, we observed an increase in the frequency of IFN-γ^+^ cells in butyrate-pretreated cell cultures as compared to control CTLs suggesting stable phenotypic changes mediated by butyrate (Supplementary Fig. 2a). Similarly, Ly5.1^+^ CTLs pretreated with butyrate and adoptively transferred into Ly5.2^+^ host mice, maintained high levels of T-bet and IFN-γ as compared to untreated Ly5.1^+^CD8^+^ T lymphocytes (Supplementary Fig. 2b).

**Figure 3:**
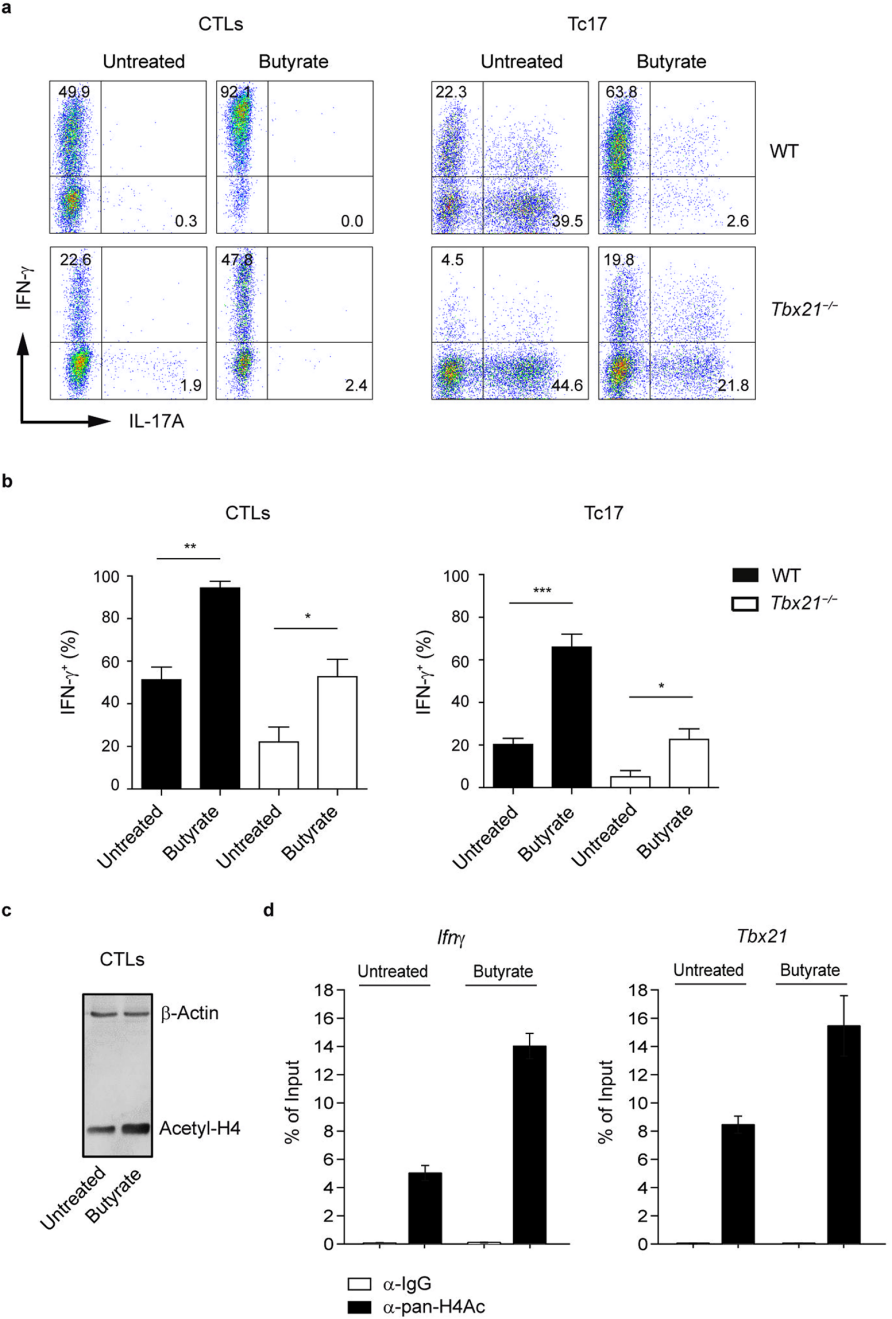
Impact of butyrate on T-bet-deficient CTLs and Tc17 cells. (**a**,**b**) Percentages of IFN-γ^+^ and IL-17A^+^ cells within CTLs and Tc17 cells derived from WT and *Tbx21*^−/−^ mice. Cells were treated with 1 mM sodium butyrate or left untreated for three days. Results (**b**) are displayed as mean ± SEM. ^*^p* < *0.05, ^**^p* < *0.01, ***p < 0.001. (**c**) CTLs were cultured for two days in the presence or absence of 1 mM butyrate. Immunoblot analysis was performed by using the antibody specific for acetylated histone H4. Three independent experiments were performed. (**d**) ChIP analysis for acetylation of histone H4 at the promoter region of *Ifn*γ and *Tbx21* genes after 24 hours of treatment of CTLs with 1 mM butyrate.

For CD4^+^ T cells, it is known that very early transcription programs govern development of specific lineages. Recently, we described an increasing heterogeneity within CD8^+^ T cells by identifying two new CD8^+^ T cell subsets, Tc17 and Tc9 cells, that might be implicated in the pathogenesis of multiple sclerosis and airway inflammation, respectively^20,21^. Interestingly, the treatment of Tc17 cells with butyrate completely altered the specific expression pattern of these lymphocytes, which is normally associated with very low amounts of granzyme B, perforin, T-bet and Eomes and high levels of IL-17A and RORγt. Upon butyrate treatment, Tc17 cells increased the levels of granzyme B, down-regulated the gene expression of Tc17-related transcription factor *Ror*γ*t* and significantly induced expression levels of CTL-associated genes *Tbx21*, *Eomes* and *Prf1* (Fig. 2c–e). To understand the molecular basis for this phenotypical switch, we treated Tc17 cells and CTLs derived from spleen and LNs of WT and T-bet deficient mice with butyrate and analyzed the expression of IFN-γ and IL-17A. In *Tbx21*^−/−^ cells, only partially defective IFN-γ production in CTLs and an incomplete switch of Tc17 cells towards the CTL phenotype was observed after three day of butyrate treatment (Fig. 3a,b). It is known that the transcription factor Eomes is essential for optimal expression of IFN-γ, granzyme B and perforin in CD8^+^ T cells^22,23^. It is conceivable that not only T-bet but also butyrate-mediated induction of Eomes might be needed for phenotypical alterations within CD8^+^ T cell subsets.

### SCFA-receptors GPR41 and GPR43 are not involved in the butyrate-mediated regulation of CD8^+^ T lymphocytes

SCFA-promoted effects on cells include both, the interaction with their specific cell surface receptors and intracellular inhibition of HDAC enzymes, thereby impacting significantly on the gene expression. SCFAs are able to directly activate their receptors GPR41 and GPR43 on intestinal epithelial cells and lymphocytes, which leads to activation of mitogen-activated protein kinase signaling, inhibition of NF-κB signaling cascades, rapid production of cytokines and chemokines and increased cell death^15,24,25^. Therefore, it is of great interest to examine the cellular pathways that are regulated by SCFAs and are involved in mediating immune responses. To investigate whether observed effects of butyrate on CD8^+^ T cells are mediated by activating the signaling pathways downstream of SCFA-receptors GPR41 and GPR43, we next tested if CD8^+^ T cells deficient for both receptors, GPR41 and GPR43, are able to upregulate their IFN-γ production upon butyrate treatment. No difference in frequencies of IFN-γ^+^ cells were found after three days of incubation of GPR41 and GPR43 deficient CTLs or Tc17 cells with butyrate as compared to butyrate-treated WT cells (Supplementary Fig. 3a–c). Similarly to WT mice, *in vivo* treatment of *Ffar2*^−/−^*Ffar3*^−/−^ mice (deficient for GPR41 and GPR43) revealed an increase in percentage of IFN-γ^+^CD8^+^ T cells in mLNs (Supplementary Fig. 3d). Together, these data show that surface receptors GPR41 and GPR43 are dispensable for butyrate-mediated effects on CD8^+^ T cells.

### HDAC-inhibitory activity of butyrate enhances the expression of CTL-associated effector molecules

**Figure 4:**
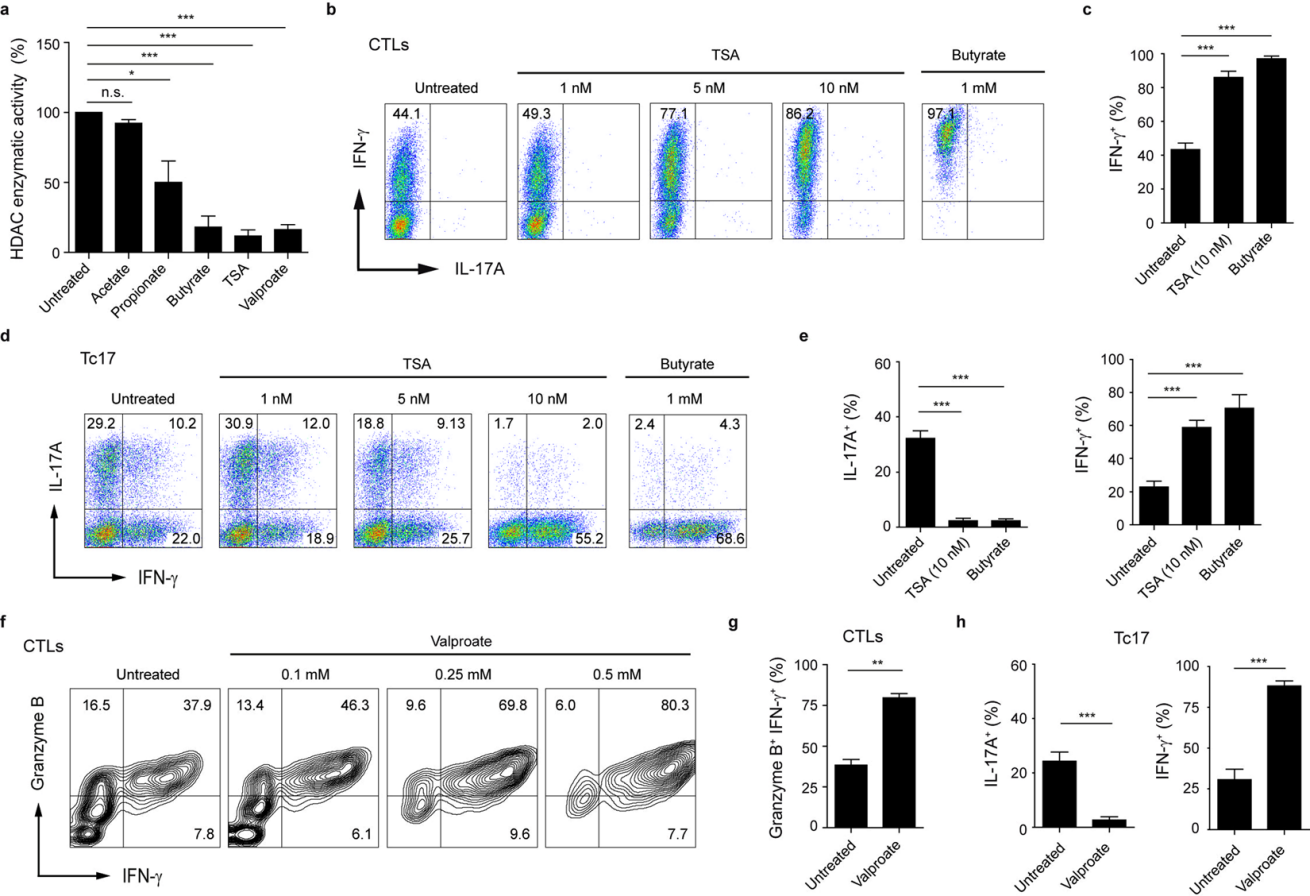
HDAC-inhibitory activity of butyrate promotes IFN-γ production in CTLs and Tc17 cells. (**a**) Impact of SCFAs on HDAC enzymatic activity in CTLs. 5 mM SCFAs or 500 nM TSA were added to CTL-derived cell lysates for 15 minutes. Fluorometric HDAC activity assay was performed as described in the section Methods. (**b**,**c**) Frequency of IFN-γ^+^ CTLs treated with indicated concentrations of TSA or sodium butyrate (1 mM). (**d**,**e**) CD8^+^ T cells were cultured under Tc17-inducing conditions and treated with increasing concentrations of TSA. Tc17 cells treated with 1 mM butyrate served as a control. (**f**,**g**) CTLs were treated with increased valproate concentrations (in **g**, 0.5 mM valproate is shown) for three days and the percentage of granzyme B^+^ IFN-γ^+^ cells was determined by flow cytometry. (**h**) Tc17 cells treated with 1 mM valproate for three days were analyzed for IL-17A and IFN-γ expression. The percentage of IFN-γ^+^ and IL-17A^+^ cells was measured by FACS analysis. (**a**,**c**,**e**,**g**,**h**) Results are pooled from three experiments. Data are expressed as mean ± SEM. n. s. = not significant, *p* < *0.05, ^**^p* < *0.01, ^***^p < 0.001.

Butyrate is known to induce histone hyperacetylation through HDAC inhibition^26^. Western blot analysis of acetylated histone H4 revealed that butyrate induced a significant increase in H4 acetylation in CTLs (Fig. 3c). We next investigated histone H4 acetylation directly at the *Tbx21* and *Ifn*γ loci in murine CD8^+^ T cells after butyrate treatment by the quantitative ChIP analysis. Our results demonstrate that butyrate increases the histone H4 acetylation of *Tbx21* and *Ifn*γ promoters in CTLs (Fig. 3d). To further test the impact of butyrate on HDAC activity of CD8^+^ T cells, we used the fluorometric HDAC activity assay for SCFA-treated cell lysates. Butyrate as well as the pan-HDAC inhibitors trichostatin A (TSA) and valproate exerted strong HDAC inhibitory activity, while acetate was not capable of blocking the enzymatic activity of CD8^+^ T cell-derived HDACs (Fig. 4a). Furthermore, butyrate was also able to strongly inhibit HDACs in cell lysates derived from *Ffar2*^−/−^*Ffar3*^−/−^ CTLs (Supplementary Fig. 3e). Importantly, when we compared the impact of TSA and valproate on IFN-γ and IL-17A expression in CTLs and Tc17 cells, we found similar expression pattern as for butyrate-treated cells. TSA- and valproate-treated Tc17 cells were capable of increasing their IFN-γ production and of switching towards CTL phenotype. Similarly, the frequency of IFN-γ^+^ or granzyme B^+^ IFN-γ^+^ cells was increased in TSA- and valproate-treated CTLs in comparison to untreated cells (Fig. 4b–h). These results suggest that increased histone acetylation mediated by physiological HDAC inhibitor butyrate might play an important role in rapid and selective induction of CTL-associated genes in CD8^+^ T cells. Recently, an increase in Foxp3^+^CD4^+^ Treg differentiation was observed upon butyrate treatment^8–10^. In the immunosuppressive microenvironment of ovarian and colorectal cancer, a high prevalence of unusual Foxp3^+^CD8^+^ regulatory T cell has been described^27,28^. Further, the expansion of Foxp3^+^CD8^+^ T cells with suppressive potential has been detected in HIV-1-infected humans^29^. Interestingly, CD8^+^ T cells cultured under Treg-inducing conditions and incubated with butyrate or TSA induced a phenotypical switch towards CTLs, associated with increased expression of T-bet and IFN-γ. This molecular shift was partially dependent on T-bet as *Tbx21*^−/−^ cells were not able to downregulate their Foxp3 expression in contrast to WT lymphocytes (Supplementary Fig. 4a–d). Previously, a molecular switch from Th2 to Th1 cell phenotype by restoring the histone acetylation at the *Ifn*γ and *Tbx21* loci was observed for human T cells treated with HDAC inhibitors^26^. Together, our novel findings indicate that in CD8^+^ T cells, the butyrate-triggered increase in IFN-γ expression is mediated through HDAC-inhibitory activity and not by engaging GPR41 and GPR43 receptors.

### Butyrate increases IFN-γ expression in CD8^+^ T cells in a dose-dependent manner

**Figure 5:**
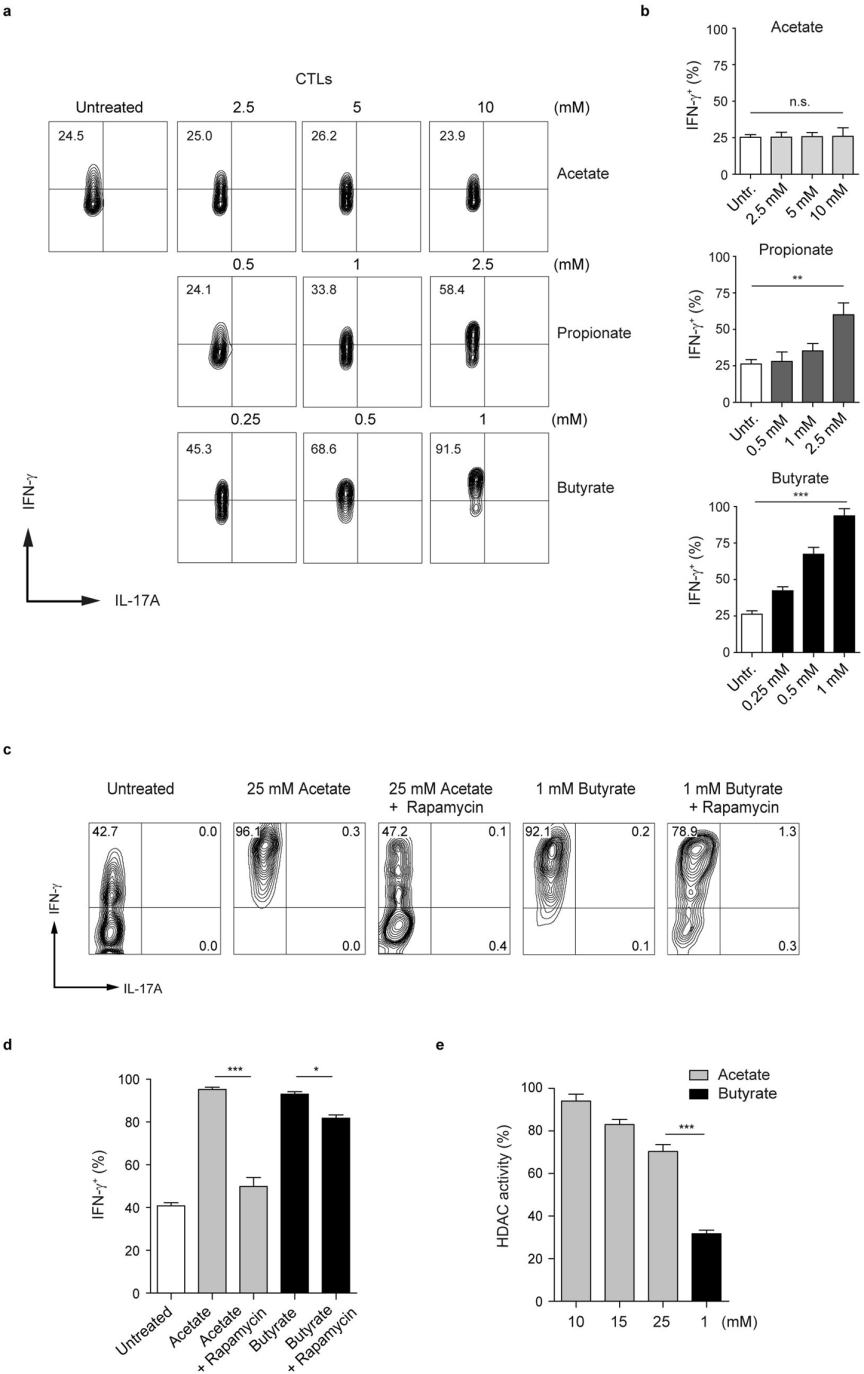
Dose-dependent impact of SCFAs on IFN-γ expression in CTLs. (**a**,**b**) CTLs were treated with increasing concentrations of SCFAs for three days. Flow cytometry analysis was used to determine percentages of IFN-γ^+^ and IL-17A^+^ cells within CTLs. Three experiments were performed. (**c**,**d**) CTLs were treated with 25 mM acetate or 1 mM butyrate in the presence or absence of rapamycin (25 nM). The production of IFN-γ is analyzed by flow cytometry. Three experiments were performed. (**e**) CTL-derived cell lysates were assayed for HDAC activity in the presence of acetate (10, 15 and 25 mM) or butyrate (1 mM). The data were compared to the HDAC activity of untreated CD8^+^ T cells, which was arbitrary set at 100 percent. Three experiments were performed. Data (**b**,**d**,**e**) are expressed as mean ± SEM. n. s. = not significant, *p* < *0.05, **p* < *0.01, ***p < 0.001.

Various cells were shown to be susceptible to apoptosis induced by butyrate and pentanoate^30,31^. We found that butyrate, and, to a lesser extent, propionate induced apoptosis in CTLs at the concentration of 5 mM. In contrast, CD8^+^ T cells treated with acetate were resistant to apoptosis even at a concentration of 10 mM (Supplementary Fig. 5a,b), indicating that acetate is less toxic when compared to other SCFAs. Based on the results related to the susceptibility to apoptosis, we assessed if SCFAs exhibit a broad concentration-dependent effect on CTLs. Cells were exposed to a wide range of SCFA concentrations, and subsequently the expression of IFN-γ and IL-17A was measured by flow cytometry. Lower butyrate concentrations (starting at 0.25 mM**)** were able to increase the production of IFN-γ in CTLs while, at the concentration of 10 mM, acetate did not exhibit any effect on effector cytokines (Fig. 5a,b). Intestinal lumen concentrations as well as blood levels of acetate in the body are much higher than that of butyrate^1^. As acetate-treated CTLs were not capable of undergoing apoptosis, we included higher acetate concentrations (>10 mM) in the flow cytometry analysis. Notably, we found that at the concentration of 25 mM, acetate-mediated effects on CTLs were similar to that of 1 mM butyrate (Fig. 5c,d). As previously described for CD4^+^ T cells and B lymphocytes^11,32^, the treatment of CTLs with acetate was associated with heightened activity of AKT/mTOR pathway (data not shown). Rapamycin, the inhibitor of mTOR complex, was able to down-regulate the expression of IFN-γ in acetate-treated CTLs to the levels detected in control lymphocytes. In contrast, the treatment of butyrate-treated CTLs with rapamycin did not strongly affect the increased production of IFN-γ (Fig. 5c,d). By comparing the HDAC inhibition mediated by 25 mM acetate and 1 mM butyrate, we observed that butyrate (at a 25-fold lower concentration as compared to acetate) had much higher HDAC-inhibitory activity (Fig. 5e). Together, there seem to exist two mechanisms through which SCFAs are capable of modulating the functional properties of CD8^+^ T cells. Our data demonstrate that butyrate, and less strongly propionate, inhibit activity of HDACs, thereby increasing the expression of IFN-γ in CD8^+^ T lymphocytes. Additionally, acetate enhances IFN-γ production likely by acting as a metabolic substrate for CTLs. This observation is in line with a recent paper that has shown that in CD8^+^ T cells, acetate was able to enhance glycolysis and cytosolic acetyl-CoA levels, which resulted in increased functional activity of memory T cells^33^.

## Discussion

The SCFAs acetate, propionate and butyrate are generated in human intestine by bacterial fermentation of undigested dietary fiber. Supplementation of diet rich in dietary fiber increases the intestinal levels of SCFAs and protects mice from allergic or autoimmune diseases^4–6^. SCFAs appear to impact on immune cells by manipulating their metabolic status, by activating or suppressing diverse signaling pathways and by inducing epigenetic changes. Hence, the scientific interest in better understanding the underlying mechanism on how SCFAs modulate immune responses has recently increased in order to develop novel therapeutic applications. Previously, it was shown that aerobic glycolysis was required for optimal INF-γ production by T cells. Interestingly, T lymphocytes prevented from engaging glycolysis were markedly compromised with regard to IFN-γ production^34^. Accordingly, we observed that the treatment of T cells with 2-deoxy-D-glucose (2-DG), an inhibitor of glycolysis, resulted in reduced IFN-γ production (data not shown). Additionally, our novel data suggest a strong involvement of epigenetic mechanisms in the modulation of IFN-γ expression since HDAC inhibition, mediated by butyrate or pan-HDAC inhibitors TSA and valproate, led to increased production of this cytokine in CD8^+^ T cells. It is well accepted that optimizing the reactivity of CTLs might be a useful strategy for improving the efficacy of T cell-mediated anti-tumor therapy. In the presence of butyrate, CTLs upregulated expression of key effector molecules, which is needed for combating the tumors. Also, Tc17 cells, which are phenotypically distinct from CTLs, increased the expression of CTL-associated molecules upon butyrate treatment. Recently, it was shown that SCFAs could enhance IL-17A expression under Th17-inducing conditions^11^. For Tc17 cells, we observed an opposite effect of butyrate, which strongly suppressed IL-17A production and enhanced IFN-γ levels. One potential reason of this discrepancy might be the usage of different differentiation protocols. Secondly, the discrepancy might result from the specific phenotype of Tc17 cells. These lymphocytes have much higher basic levels of IFN-γ than Th17 cell. A sufficient availability of IFN-γ likely augments the butyrate-mediated phenotypic switch from Tc17 cells towards CTLs. Further, we found a significant difference among SCFAs with regard to their ability to regulate the gene expression of CTLs and Tc17 cells. The profound effect of butyrate on CD8^+^ T cells was predominantly mediated by its HDAC-inhibitory activity since butyrate induced an increase in H4 acetylation at *Tbx21* and *Ifn*γ genes. For CD4^+^ T cells, a previous study found that butyrate was able to enhance acetylation of histone H3 of *Tbx21* and *Ifn*γ promoter regions under Th1- and Th2-polarizing conditions^26^. Interestingly, acetate is not capable of acting as a strong HDAC inhibitor, but it can be efficiently metabolized by various cells. CD8^+^ T cells have been shown to functionally integrate high systemic acetate concentrations to metabolically boost memory T cell responses^33^. We observed that higher acetate concentrations increased IFN-γ production in CTLs and that this effect was dependent on activity of mTOR complex.

High-fiber diet in association with enhanced intestinal butyrate synthesis has been shown to attenuate the development of colorectal cancer associated by chronic colitis^16^. Further, butyrate can directly promote cancer cell apoptosis^35^. Here, we suggest a novel mechanism of how butyrate can exert anti-cancer effects by stimulating CD8^+^ T cells and increasing their effector functionality. While physiologically active, low butyrate amounts in the intestine induce the differentiation of Foxp3^+^ Tregs, higher concentration of this SCFA might rather promote anti-tumor effects by optimizing the effector function of CD8^+^ T cells. Of note, a very recent study has revealed that the treatment of mice with butyrate was capable of enhancing the generation of influenza-specific CD8^+^ T cells, which considerably contributed to the resolution of influenza infection^36^. In summary, natural HDAC inhibitors such as butyrate might be considered as novel therapeutic tools to modulate the function of CD8^+^ T cells and to potentiate their anti-tumor and anti-viral capacity.

## Methods

### Mice and oral administration of butyrate

WT animals as well as *Tbx21*^−/−^ and *Ffar2*^−/−^*Ffar3*^−/−^mice (all mouse strains were on C57BL/6 background) were housed in specific pathogen free (SPF) conditions at the animal facility of the Philipps-University of Marburg, Germany. WT mice were obtained from Charles River Laboratories. *Ffar2*^−/−^*Ffar3*^−/−^ mice were generously provided by Dr. Stefan Offermanns (Max Planck Institute for Heart and Lung Research, Bad Nauheim, Germany). All animal procedures were carried out according to the German animal protection law. Experiments were done after approval by Regierungspräsidium Gießen, Germany (animal experimentation application EX7-2015). In some experiments, WT mice were orally treated with 150 mM sodium butyrate for four weeks as described previously^8^. Water solutions containing butyrate were pH-matched and changed weekly.

### Isolation and *in vitro* differentiation of CD8^+^ T cells

CD8^+^ T lymphocytes were purified from spleens and LNs using the kit for negative isolation (the purity of cells was between 90–95%). In brief, spleens and LNs were harvested and disrupted to obtain single-cell suspensions. Splenic cell suspensions were treated with RBC lysis buffer before lymphocyte isolation. To generate CTLs, purified CD8^+^ cells were stimulated with 5 µg/ml plate-bound anti-CD3 (clone 145–2C11) and 1 µg/ml soluble anti-CD28 (clone 37.51) Abs in the presence of 50 U/ml rhIL-2 (Novartis) for three days. In some experiments, cells were treated with 10 µg/ml of anti-IFN-γ (clone XMG1.2) to obtain sub-optimal CTL conditions. For Tc17 cell differentiation, 1 ng/ml rhTGF-β1 (PeproTech) and 40 ng/ml IL-6 (PeproTech) together with 5 µg/ml anti-IFN-γ were added into RPMI medium during the cell stimulation. For Treg-inducing conditions, cell suspensions were treated with 2 ng/ml TGF-β1 and 100 U/ml rhIL-2 in the presence of anti-IFN-γ (5 µg/ml). Cells were treated with SCFAs and cultured at 37 °C for three days. For this purpose, 1 mM of sodium butyrate, sodium propionate or sodium acetate (all SCFAs, Sigma-Aldrich) were used.

### Adoptive cell transfer

CD8^+^ T cells were purified from spleens and LNs of Ly5.1^+^ C57BL/6 mice and were cultured under CTL-inducing conditions for three days in the presence or absence of 1 mM butyrate. 2 × 10^6^ CTLs were used for T cell adoptive transfer into Ly5.2^+^ mice. Ten days after the transfer of cells, the expression of IFN-γ and T-bet within Ly5.1^+^CD8^+^ T lymphocytes was anylazed.

### Flow cytometry and antibodies

After three days of the cell culture, CD8^+^ T cell were restimulated for 4–5 h with PMA (50 ng/mL) and ionomycin (750 ng/mL) in the presence of 5 mg/mL brefeldin A (all three chemicals, Sigma-Aldrich). Afterwards, cells were treated with fluorochrome-labeled Abs. For the cytokine staining, cells were fixed with 2% formaldehyde and permeabilized by using saponin buffer. For intracellular staining of transcription factors, the Foxp3 staining kit (eBioscience) was used. The analysis of FACS data was performed using FlowJ_V10 software (TreeStar). The following anti-mouse Abs were used: anti-IFN-γ (XMG1.2), anti-CD3 (145-2C11), anti-CD8 (53-6.7), anti-CD45.1 (A20), anti-granzyme B (16G6), anti-IL-17A (eBio17B7), anti**-**Foxp3 (FJK-16s) and anti-T-bet (eBio4B10). All Abs were purchased from eBioscience.

### Chromatin immunoprecipitation (ChIP) analysis

CTLs were cultured for 24 hours in the presence or absence of 1 mM butyrate. After 24 hours of the cell culture, ChIP-qPCR was essentially performed as described previously^37^. Briefly, after fixation, lysis, sonication, and preclearing, chromatin equivalent to 2.5 × 10^6^ nuclei per sample was precipitated with rabbit control IgG (Sigma-Aldrich I5006) or α-acetylated histone H4 (Millipore, Upstate, 06-866). After purification, DNA was amplified using the following primers: *Ifn*γ promoter fw CATACCCTTTCCTTGCTTTTC, rv TTGTGGGATTCTCTGAAAGCA; *Tbx21* (T-bet) promoter fw TGGGGCGACAAGAGACTTAC, rv GAATTCGCTTTTGGTGAGGA.

### HDAC activity assay

Fluorometric HDAC activity was measured according to the manufacturer’s protocol (Bachem). Briefly, CD8^+^ T cells were cultured under CTL-inducing conditions for three days. Subsequently, CTL-derived cell lysates were incubated with various SCFAs (5 mM) or with TSA (500 nM) for 15 minutes. In some experiments, 25 mM acetate and 1 mM butyrate were used for HDAC assay. To measure the inhibitory activity of SCFAs on HDACs, the cell lysates were treated with fluorogenic substrate Ac-Arg-Gly-Lys-AMC (Bachem) for next 30 minutes. HDAC-inhibitory activity of SCFAs was calculated relative to the enzymatic activity of untreated CTLs (this value was arbitrarily set to 100%). The measurement of fluorescence (free AMC) was carried out at the FLUOstar Omega device (BMG Labtech).

### Western blotting

Immunoblotting was performed as described previously^14^. In brief, cell lysates were generated from 2 × 10^6^ CTLs on day 2 of the cell culture. Protein samples were loaded on 10% gel for SDS-PAGE and were subsequently transferred to a PVDF membrane. Acetylated Histone H4 was detected using an anti-acetyl-histone H4 Ab (Millipore, 06-866). For detection of β-actin, a monoclonal anti-β-actin Ab (Sigma-Aldrich, AC-74) was used.

### Quantitative RT-PCR

To isolate the total RNA from cultured CTLs or Tc17 cells, the High Pure RNA Isolation Kit was used according to the manufacturer’s instruction (Roche). Following the cDNA synthesis, the relative expression of *Ifn*γ, *Ror*γ*t*, *Tbx21, Eomes* and *Prf1* was performed with specific primers using a StepOnePlus device (Applied Biosystems). The expression of all genes was normalized to the expression of the housekeeping gene *Hypoxanthine-guanine phosphoribosyl transferase (Hprt1*). Quantitative RT-PCR was performed by using the following primers: *Ifnγ* fw GCAACAGCAAGGCGAAAAAG, *Ifnγ* rv TTCCTGAGGCTGGATTCGG; *Tbx21* fw CAACAACCCCTTTGCCAAAG, *Tbx21* rv TCCCCCAAGCAGTTGACAGT; *Eomes* fw CCTTCACCTTCTCAGAGACACAGTT, *Eomes* rv TCGATCTTTAGCTGGGTGATATCC; *Prf1* fw GCAGATGAGAAGAGCATACAGGAC, *Prf1* rv TCTGAGCGCCTTTTTGAAGTC; *Rorγt* fw TCCTGCCACCTTGAGTATAGTCC, *Rorγt* rv GGACTATACTCAAGGTGGCAGGA.

### Statistical analysis

Mean values of two groups were compared by using the unpaired Student’s *t*-test. To compare multiple groups, results were analyzed using the one-way analysis of variance (ANOVA). Values of p < 0.05 were considered statistically significant. Data are typically presented as means ± SEM. For statistical analysis, the GraphPad Prism 5 software was used.

## Electronic supplementary material

Supplementary Dataset 1
