# Supplementary material for: Regulation of the effector function of CD8+ T cells by gut microbiota-derived metabolite butyrate
Source: Sci Rep. 2018 Sep 26;8:14430. doi: 10.1038/s41598-018-32860-x (PMC6158259; doi:10.1038/s41598-018-32860-x)
Supplement: Supplementary file 1 — Supplementary Dataset 1 [file 41598_2018_32860_MOESM1_ESM.pdf]

Supplementary Figures:

Regulation of the effector function of CD8$^+$ T cells by gut microbiota-derived metabolite butyrate

Maik Luu[1], Katharina Weigand[1], Fatana Wedi[1], Carina Breidenbend[1], Hanna Leister[1], Sabine Pautz[1,2], Till Adhikary[3], Alexander Visekruna[1]

[1]Institute for Medical Microbiology and Hygiene, Philipps University of Marburg, Marburg, Germany.
[2]Department of Biochemistry, University of Kassel, Kassel, Germany.
[3]Institute of Molecular Biology and Tumor Research (IMT), Center for Tumor- and Immunobiology, Philipps-University Marburg, Marburg, Germany.

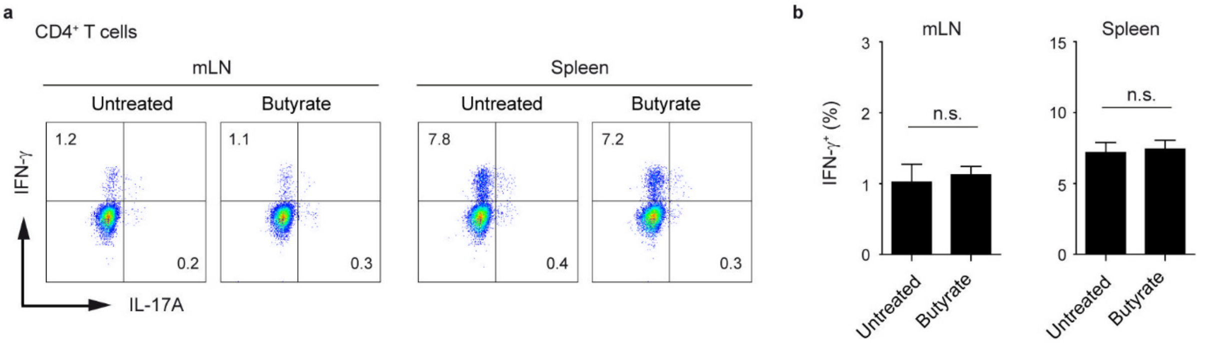

Supplementary Figure 1. Oral treatment of WT mice with butyrate. (**a**) Frequency of IFN-γ^+ and IL-17A$^+$ CD4$^+$ lymphocytes in the spleen and mLNs four weeks after oral treatment of WT mice with 150 mM butyrate. Two experiments with five mice per group were performed. A representative of two experiments is shown. Error bars (**b**) indicate SEM; n. s. = not significant.

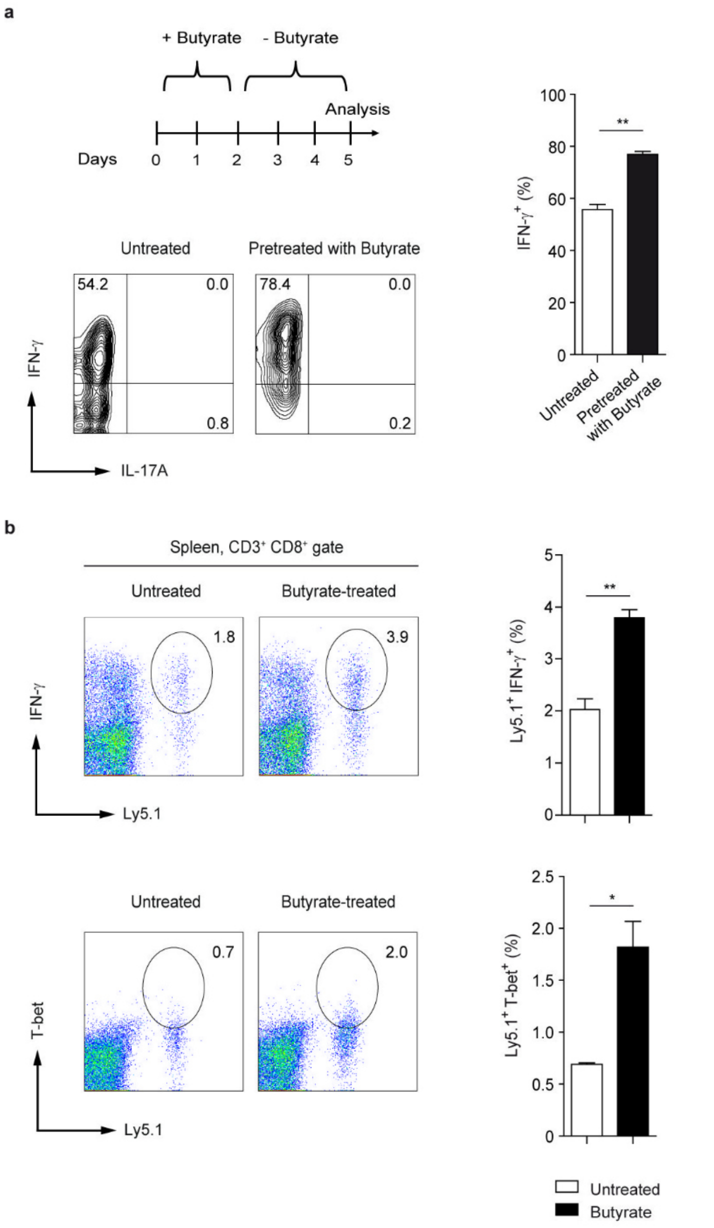

Supplementary Figure 2. Stability of butyrate-mediated phenotype in CTLs. (**a**) CTLs were pretreated with 1 mM butyrate. After two days of the cell culture, butyrate was removed from the supernatant and subsequently cells were cultured for next three days in the absence of butyrate. On day 5 of the cell culture, frequencies of IFN-γ^+ and IL-17A$^+$ cells were measured by flow cytometry. Three experiments were performed. Data (right) are expressed as mean ± SEM. **p < 0.01. (**b**) Ly5.1$^+$ CTLs were cultured for three days in the presence or absence of 1 mM butyrate. 2 x 10^6 CTLs were adoptively transferred into Ly5.2$^+$ mice and ten days later, the frequency of splenic Ly5.1$^+$IFN-γ^+ (upper panel) and Ly5.1$^+$T-bet$^+$ cells (lower panel) was analyzed within CD3$^+$CD8$^+$ gate. Two experiments were performed. Results (right) are expressed as mean ± SEM. *p < 0.05, **p < 0.01.

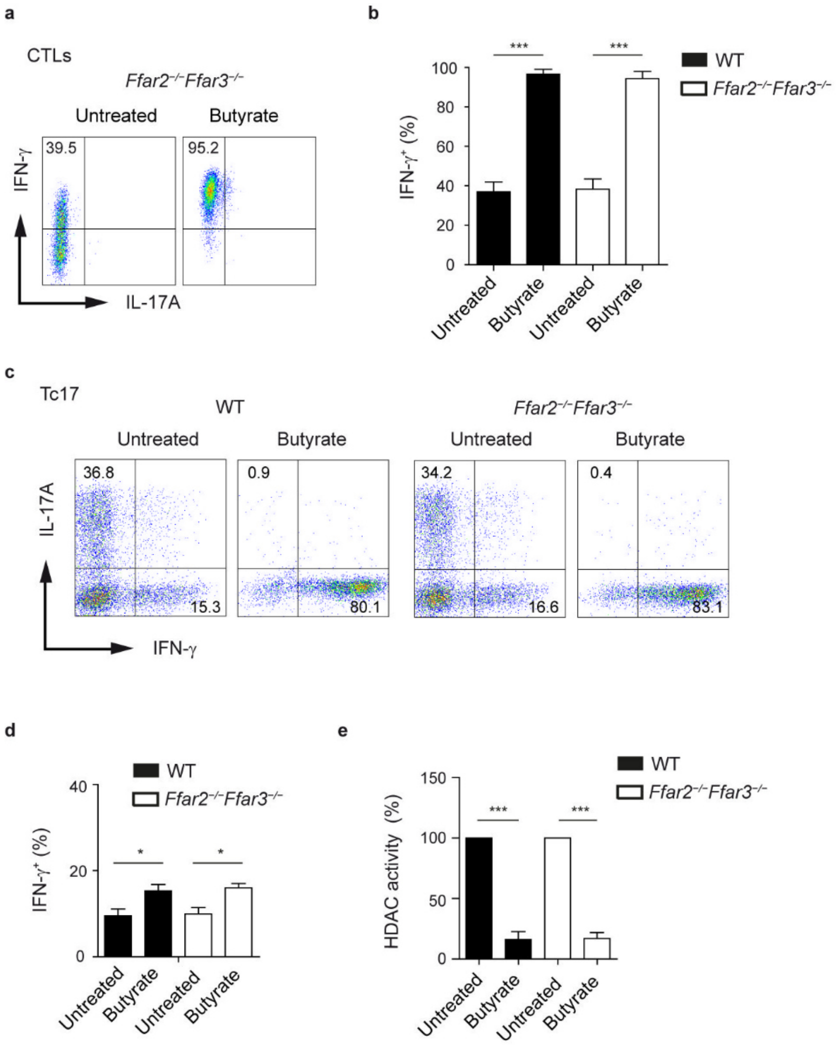

Supplementary Figure 3. Butyrate-mediated effects on $CD8^+$ T cells are independent of GPR41 and GPR43. (**a-c**) Frequency of IFN-γ^+ and IL-$17A^+$ cells within CTLs and Tc17 cells deficient for SCFA receptors GPR41 and GPR43. Cells were treated with 1 mM sodium butyrate for three days. As a control, WT CTLs (**b**) and Tc17 cells (**c**) were used. Three similar experiments were performed. (**d**) *Ffar2*$^{-/-}$*Ffar3*$^{-/-}$ and WT mice (n = 5 mice per group) were orally treated with 150 mM butyrate for four weeks. Frequency of IFN-γ^+CD8^+ T cells in mLNs was determined by FACS analysis. (**e**) HDAC activity was performed on the cell lysates derived from *Ffar2*$^{-/-}$*Ffar3*$^{-/-}$ and WT CTLs in the presence or absence of 5 mM butyrate. Results (**b**, **d** and **e**) are expressed as mean ± SEM. $^*p < 0.05$, $^{***}p < 0.001$.

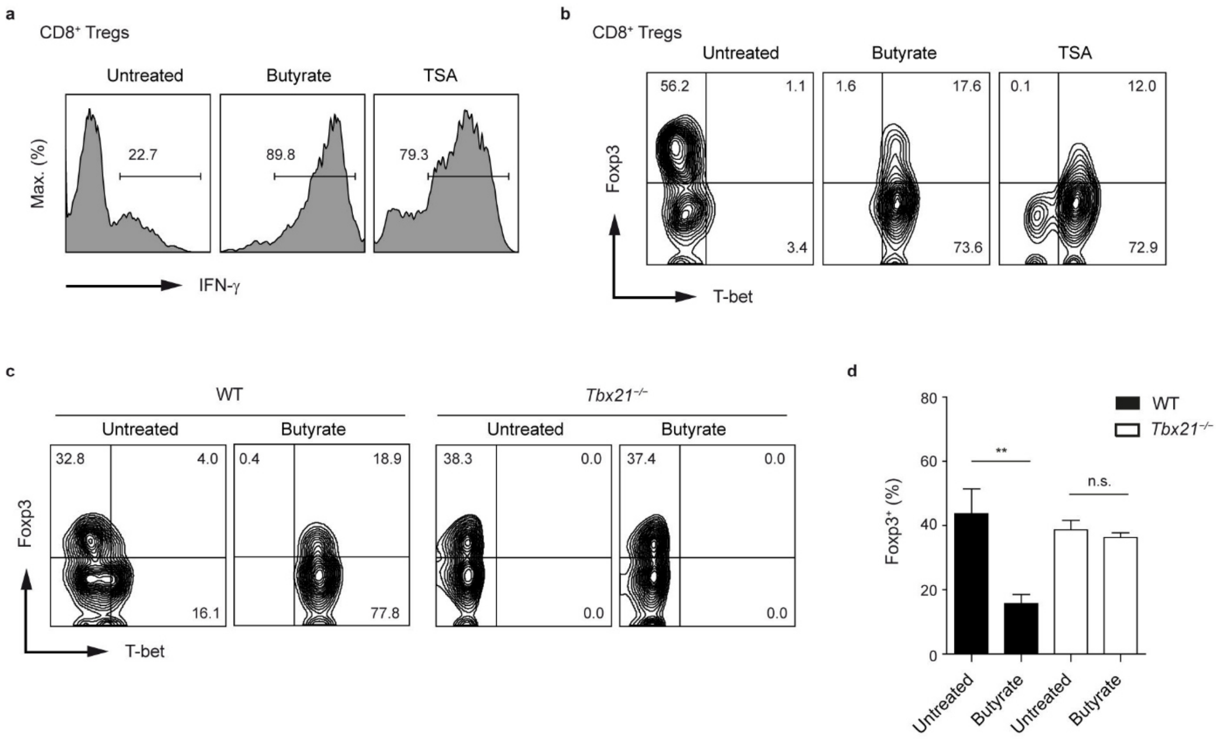

Supplementary Figure 4. Impact of butyrate and TSA on $CD8^+$ T cells cultured under Treg-inducing conditions. (**a** and **b**) WT $CD8^+$ T cells were cultured under Treg-inducing conditions for three days in the presence of 1 mM sodium butyrate or 10 nM TSA. The frequency of IFN-γ^+ (**a**), or $Foxp3^+$ and T-bet^+ (**b**) cells within $CD8^+$ Tregs was analyzed by flow cytometry. Representatives of three similar experiments are shown. (**c** and **d**) $CD8^+$ T cells derived from WT and T-bet-deficient mice were cultured under Treg-inducing conditions for three days. On day three of the cell culture, the intracellular staining for Foxp3 and T-bet was performed. Results (**d**) are expressed as mean ± SEM. n. s. = not significant, $^{**}p < 0.01$.

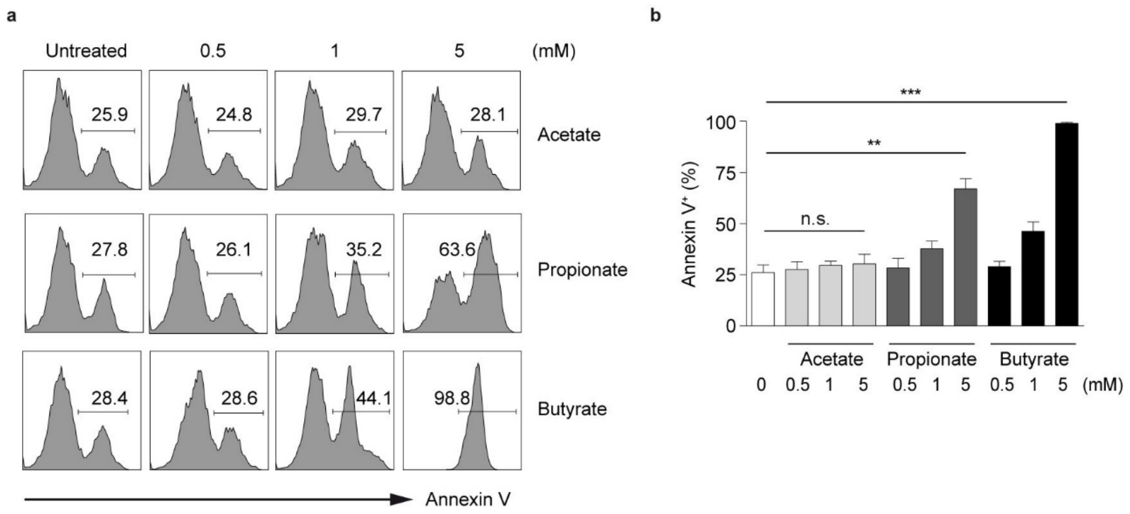

Supplementary Figure 5. Butyrate and propionate increase apoptosis in $CD8^+$ T cells. (**a** and **b**) WT CTLs were cultured for three days in the presence of increasing SCFA concentrations. The frequency of annexin V^+ cells was analyzed by flow cytometry. A representative of three experiments is shown (**a**). Results (**b**) are expressed as mean ± SEM. n. s. = not significant, $**p < 0.01$, $***p < 0.001$.

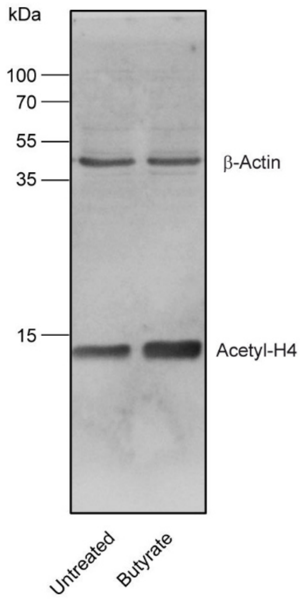

Supplementary Figure 6. Original full-length blot of data shown in Figure 3C**.** Acetyl-H4 and β-Actin signals were detected on the same blot membrane. PageRuler Prestained Protein Ladder (Thermo Scientific) was used to determine the size of stained proteins.
